# Coronary atherosclerosis profile in patients with end-stage liver disease prior to liver transplantation due to alcoholic fatty liver: a coronary CTA study

**DOI:** 10.1007/s00330-020-07037-8

**Published:** 2020-08-04

**Authors:** Fabian Steinkohl, Fabian Barbieri, Thomas Senoner, Sylvia Strobl, Armin Finkenstedt, Fabian Plank, Christian Langer, Christoph Beyer, Katharina Birkl, Gerlig Widmann, Heinz Zoller, Wolfgang Dichtl, Guy Friedrich, Herbert Tilg, Gudrun Feuchtner

**Affiliations:** 1 Dept. Radiology, Medical University of Innsbruck, Anichstr. 35, A-6020 Innsbruck, Austria; 2 Radiology, St. Vincent Hospital Zams, Zams, Austria; 3 Dept. Internal Medicine III, Cardiology, Medical University of Innsbruck, Innsbruck, Austria; 4 Dept. Internal Medicine I, Gastroenterology, Hepatology and Endocrinology, Medical University of Innsbruck, Innsbruck, Austria

**Keywords:** Angiography, computed tomography, Coronary artery disease, End-stage liver disease, Alcohol abuse, Risk factors

## Abstract

**Objectives:**

To assess the coronary atherosclerosis profile by coronary computed tomography angiography (CTA) in patients with end-stage liver disease (ESLD) due to alcohol-related liver disease (ARLD) evaluated for liver transplantation (LT), in a retrospective matched case-controlled cohort study.

**Methods:**

One hundred forty patients (age 60.6 years ± 9.8, 20.7% females) who underwent coronary CTA were included. Seventy patients with ESLD due to ARLD (ESLD-alc) were propensity score (1:1) matched for age, gender, and the major 5 cardiovascular risk factors with healthy controls. CTA analysis included the following: stenosis severity according to CAD-RADS as (0) = no, (1) minimal < 25%, (2) mild 25–50%, (3) moderate 50–70%, and (4) severe > 70% stenosis, total mixed plaque burden weighted for non-calcified component (G-score) and high-risk plaque criteria (Napkin-Ring, low attenuation plaque, spotty calcification, positive remodeling).

**Results:**

Prevalence of coronary artery disease (CAD) was high (84.4%) in the ESLD-alc group but similar to controls. Stenosis severity was similar (CAD-RADS, 1.9 vs. 2.2, *p* = 0.289). High-grade stenosis (> 70%) was observed in 12.5% of ESLD-alc patients. High-risk plaques were less frequent in the ESLD-alc cohort as compared to controls (4.5% vs. 37.5%, *p* < 0.001), and total mixed plaque burden was lower (G-score, 4.9 versus 7.4, *p* = 0.001). Plaque density was lower in controls (56.6HU ± 3.2 vs. 91.3HU ± 4.5, *p* = 0.007) indicating more lipid-rich in controls, but higher mixed fibro-calcific plaque component in those with alcohol-related ESLD.

**Conclusion:**

Patients with alcohol-related ESLD exhibit more mixed fibro-calcified plaques but less plaque with high-risk features and less fibro-fatty plaque burden, while total CAD prevalence is high.

**Key Points:**

*• Patients with ESLD prior to LT have a high total prevalence of CAD and stenosis severity, which is similar to those of healthy controls with an identical cardiovascular risk profile.*

*• Patients with ESLD prior to LT due to alcohol abuse have more calcific but less fibro-fatty plaque and less high-risk plaque.*

*• CTA seems to be a useful imaging technique for risk stratification prior to LT.*

## Introduction

Cardiac complications are a frequent cause of death after liver transplantation (LT) [1]. Therefore, preoperative cardiac risk stratification is needed in patients, who are listed for LT. It seems that coronary calcium score (CCS) [2] and coronary computed tomographic angiography (CTA) are safe and useful tools for this task [3].

It is known that moderate amounts of alcohol have protective effects on coronary artery disease (CAD) [4]. Heavy drinking may adversely affect the cardiovascular system, but there is controversy in the literature [5, 6]. Patients with end-stage liver disease (ESLD) due to excessive alcohol consumption form a large cohort of all LT patients. The direct effects of excessive alcohol consumption on the coronary atherosclerosis profile in these patients prior to liver transplantation have not yet been investigated in a study using coronary computed tomography angiography (CTA).

Coronary CTA is a highly accurate quantification tool for coronary atherosclerosis in terms of stenosis severity (CAD-RADS) [7] and total (including non-calcified and mixed) plaque burden, and enables the characterization of “high-risk” vulnerable plaque features, such as the napkin-ring sign (NRS), low attenuation fibro-fatty plaque (LAP), spotty calcification, and positive remodeling. These “high-risk” plaque markers have recently emerged as indicators for increased risk of major cardiovascular events (MACEs) [8–10].

Therefore, the purpose of our study was to investigate coronary atherosclerosis characteristics in patients with ESLD due to alcohol-related liver disease (ARLD) and a documented history of alcohol abuse (ESLD-alc) [11], in a retrospective matched case control cohort study.

## Methods

### Study population

All patients with ESDL referred for CAD screening prior to LT were extracted from our coronary CTA database consisting of patients referred to coronary CTA between 2006 and 2016. Then, patients with a history of an alcohol use disorder [12] documented in hospital information system were included into the “ESLD-alc” group. Those were propensity score (1:1) matched with non-regular alcohol drinking individuals without known liver disease.

Conventional coronary risk factors according to standardized European Society of Cardiology (ESC) criteria were collected: arterial hypertension (systolic blood pressure > 140 mmHg or diastolic blood pressure > 90 mmHg), dyslipidemia (total cholesterol > 200 mg/dl or HDL < 40 mg/dl), family history (myocardial infarct or sudden cardiac death in an immediate male relative < 55 years or female < 65 years), smoker (current or quit within the last 6 months), and diabetes.

The new model for end-stage liver disease (MELD) score was calculated as follows: 9.57 × ln (creatinine mg/dl) + 3.78 × ln (total bilirubin mg/dl) + 11.2 × ln (INR) + 6.43, [11]. The Child-Pugh Stadium was recorded and classified into classes A, B, and C.

### Inclusion criteria

#### ESLD-alc group

- Evidence of alcohol abuse reported during psychiatric LT evaluation and/or liver histopathology (Mallory Weiss bodies).
- Meeting ESLD criteria [11] and fulfilling criteria for LT listing (MELD ≥ 15 or clinical signs of decompensated liver cirrhosis like refractory ascites, hepatic encephalopathy, sarcopenia). Diagnosis of ARLD was made according to the current European guidelines [13].
- All patients were evaluated prior to LT.

#### Control group

- Patients denied alcohol consumption by a questionnaire prior to CTA exam, or reported non-regular occasional drinking of maximum 1 glass of an alcoholic beverage maximal once per week.
- Normal liver enzymes.

### Exclusion criteria for coronary CTA

- Renal dysfunction (GFR < 30 ml/min)
- Previous coronary artery bypass grafting (CABG) grafting
- Current acute coronary syndrome (ACS) or unstable angina
- Severe aortic stenosis

### Coronary computed tomography

#### Coronary computed tomography angiography

Non-contrast ECG-gated CCS with standardized scan parameters (detector collimation 64 × 1.5 mm; 120 kV) was performed. The Agatston Score was calculated [14]. Then, coronary CTA was performed with a retrospectively ECG-gated 64-slice CTA (*GE Healthcare or Siemens Somatom Sensation 64*) from 2005 to 2009, and from 2010 onwards with a 128-slice dual source CTA (*Definition FLASH, Siemens*) with a detector collimation of 2 × 64 × 0.6 mm, a *z*-flying spot of 64 × 0.6 mm, and a rotation time of 0.28 s. Prospective ECG triggering was used in regular heart rates < 65 bpm (diastolic padding, 70% of RR interval); in heart rates > 65 bpm and irregular heart rhythm, retrospective ECG-gating was applied.

An iodine contrast agent (*iopromide*, *Ultravist 370*, *Bayer Pharma AG*) was injected intravenously (flow rate 4–6 ml/s followed by 40-ml saline chaser), triggered into arterial phase (bolus tracking; 100 HU threshold; ascending aorta). Contrast volume ranged from 65 to 120 ml, and was adjusted to the individual patient characteristics using a standardized scheme. Axial images were reconstructed with 0.75-mm slice width (increment 0.4/medium-smooth kernel B26f) during best diastolic and systolic phase. The same CT protocol was used for both groups.

#### CTA image analysis

Curved multiplanar reformations (cMPRs) and oblique interactive MPR of all vessels using 3-D post-processing software (*SyngoVia™, Siemens Healthineers*) were generated:

1. *Coronary stenosis severity* was scored as follows: minimal < 25%, mild ≤ 25–49%, intermediate 50–69%, or severe ≥ 70% according to CAD-RADS [15] per coronary segment (American Heart Association (AHA)-modified-16-segment classification) [16].
2. The coronary *plaque types* were characterized as: calcified (T1), mixed (dominantly calcified > non-calcified) (T2), mixed (dominantly non-calcified > calcified) (T3), non-calcified (T4) per coronary segment. Calcified and non-calcified plaque components were defined as hyper- and hypoattenuating lesions with > and < 150 HU [10]. The *G-score* [17] (= sum of plaque types T1–T4 for each segment), as radiologic marker for an increased non-calcifying plaque burden, per coronary segment (AHA-modified-16-segment classification) was calculated.
3. Quantitative *High-risk plaque* (*HRP*) *analysis* [8]

- *Non-calcified plaque* (*NCP*) was defined as being hypodense as compared to vessel lumen, < 150 HU [18]. CT density was screened by utilizing “pixel-lens” [16] and the minimal HU was recorded. Then, an area ROI was placed into the lowest density plaque area, and drawn as large as possible, while sparing areas affected by artifacts.
- *Low attenuation plaque* (*LAP*) < 60HU was defined as fibro-fatty plaque and regarded as HRP criterion [10].
- *Napkin-ring sign* (*NRS*) was defined as hyperdense rim surrounding a hypodense plaque core [9].
- *Spotty calcification* was defined as calcification < 3-mm size within a NCP.
- The *remodeling index* (*RI*) was calculated as the ratio of the maximal cross-sectional vessel diameter including the plaque and the lumen, and its closest proximal (or in ostial lesions distal) normal reference vessel lumen diameter. An RI was defined as positive if > 1.1.

If a patient had multiple lesions, all lesions were quantified separately and counted.

A high-risk plaque was defined as such: if a minimum of 2 of 4 criteria were present [10].

Coronary CTA image analysis was performed by one observer (> 10 years’ experience) and a second independent observer with > 6 months of training. Consensus reading was obtained.

### Statistical analysis

Statistical analysis was performed using IBM SSPS™ software (*V 25.0, IBM Corporation*). Propensity score matching was performed by 1:1 method for age, gender, body mass index (BMI), and the major 5 risk factors (arterial hypertension, smoking, positive family history, dyslipidemia, and diabetes).

Quantitative variables were expressed as mean ± standard deviation (SD); categorical variables reported as absolute values and percentages. Normal distribution was tested with Kolmogorov-Smirnov test.

Mean differences between parametric data were tested by using the appropriate tests according to their distribution (*t* test for normally, and Mann-Whitney *U* for non-normally distributed and rank-scale data such as G-score, segment involvement score, CAD-RADS, and CCS). Differences in categorical data (gender, coronary risk factors, prevalence of CAD, CAD-RADS 0–4, high-risk plaque) between groups were determined with chi-square or Fisher’s exact test (if *n* ≤ 5 per group). A *p* value of less than 0.05 was considered significant.

## Results

A total of 488 patients with ESLD listed for LT were extracted from our coronary CTA database consisting of 9988 patients. Then, those patients with a history of alcohol abuse were selected (*n* = 97) and propensity score matched 1:1 with patients from our database consisting of 4400 patients, who denied regular alcohol consumption. Seventy-two patients with ESLD due to alcohol abuse could be matched and were enrolled. Two patients from each group were excluded; due to either inadequate CT image quality (*n* = 2) (high image noise or motion artifacts), or other exclusion criteria (severe aortic stenosis (*n* = 1) or previous CABG grafting (*n* = 1)).

In total, 140 patients (70 with ESLD-alc and 70 controls) were included. Sixty-four underwent both coronary CTA and CCS. In 6 patients, only CCS was performed due to renal dysfunction.

Demographic and clinical characteristic are shown in Table 1. There was no significant difference with regard to age, gender, and the major five cardiovascular risk factors. The prevalence of previous acute renal injury was higher in ESLD as compared to controls. Left ventricular ejection fraction (LVEF) was normal in both groups; none of the subjects had severe heart failure with a LVEF of less than 35%.

**Table 1:** Study cohort

	ESLD-alc *N* = 64	CR *N* = 64	*p* value
Age (years)	60.8 ± 9	60.5 ± 10	0.850
Females	8 (12.5%)	9 (14.1%)	> 0.999
BMI kg/m^2^	26.3 (± 37)	26.4 (± 43)	0.609
Arterial HT	57 (89%)	60 (93.7%)	0.528
Smoking	36 (56.2%)	34 (53.1%)	0.859
Positive FH	2 (3.1%)	3 (4.6%)	> 0.999
Dyslipidemia	7 (10.9%)	8 (12.5%)	> 0.999
Diabetes	9 (14.0%)	8 (12.5%)	> 0.999
MELD score	15.7 ± 6.0		
Child-Pugh stage			
A	9 (14.1%)		
B	34 (53.1%)		
C	21 (32.8%)		
Previous acute renal injury	12 (18.7%)	0 (0%)	
LV EF %	59.9 ± 6.1	63.6 ± 12.8	
EF < 35%	0 (0%)	0 (0%)	n.s.

*Abbreviations*: *EF* = ejection fraction. *LV* = left ventricle. *MELD* = model for end-stage liver disease. *HAT* = hypertension. *FH* = family history. *BMI* = body mass index. *n.s.* = non-significant

Table 2 shows coronary CTA results. Prevalence of CAD, defined as presence of plaque, was high but comparable between the ESLD-alc group and controls (84.4% vs. 87.5%), as well as stenosis severity (CAD-RADS), which was similar between the two groups (1.9 vs. 2.2, *p* = 0.289). The prevalence of high-grade > 70% stenosis was 12.5% in ESLD-alc patients and did not differ significantly from controls.

**Table 2:** Coronary atherosclerosis profile by coronary CTA: 140 patients (*n* = 128 with CTA and CCS, *n* = 12 with coronary calcium score (CCS) only)

	ESLD-alc *N* = 64	controls *N* = 64	*p* value
CAD *n* (%)	54 (84.4%)	56 (87.5%)*	0.465*
CAD-RADS 1–2	32	35	
CAD-RADS 3	13	10	
CAD-RADS 4	8 (12.5%)	12 (18.7%)	
CAD-RADS	2 (IQR 1)	2 (IQR 2)	0.289**
G-score	8 (IQR 7)	4 (IQR 7)	0.001**
HRP *n* (%) *	5 (4.5%)	24 (37.5%)	< 0.001*
#HRP total (*n*)	6	35	< 0.001*
LAP density (HU)	91.3 ± 4.5	56.6 ± 3.2	0.007***
Coronary Calcium Score (AU) *N* = 70	107 (IQR 1046)	95 (IQR 403)	0.673**
NCP in Calcium Score zero	4/13 (30.7%)	10/17 (58.8%)	0.551*

*Abbreviations*: *LAP* = low attenuation plaque. *NCP* = non-calcified plaque. *AU* = Agatston Units. *N* = counts. *HU* = Hounsfield Units. *ESLD-alc* = end-stage liver disease due to alcohol consumption

*per patient. #HRP = number of HRP. *Chi-square; **Mann-Whitney *U* (non-parametric); ***independent *t* test

Counts are displayed as numbers (N). For CADRADS, G-score, and CCS, the median and IQR (interquartile range) are shown

**Fig. 1:**
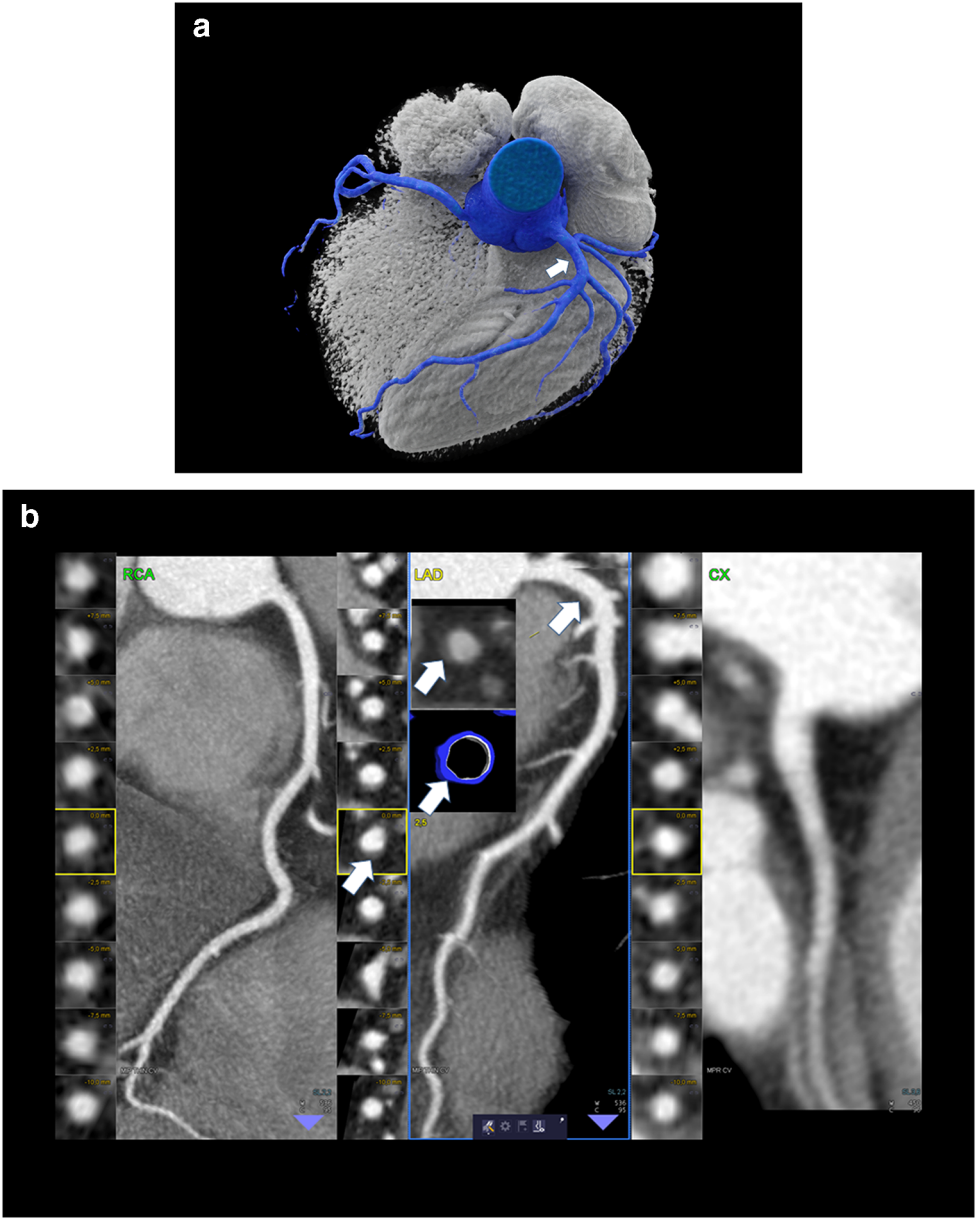
A 64-year-old male with one risk factor (arterial hypertension) and ESLD due to alcohol-related liver disease. CTA shows a small non-calcified plaque in the proximal LAD (arrow) with a high plaque density (HU 80–90) and minimal stenosis (CAD-RADS 1). Calcium score was zero. VRT (left) and cMRP (right) of the RCA; LAD and CX

**Fig. 2:**
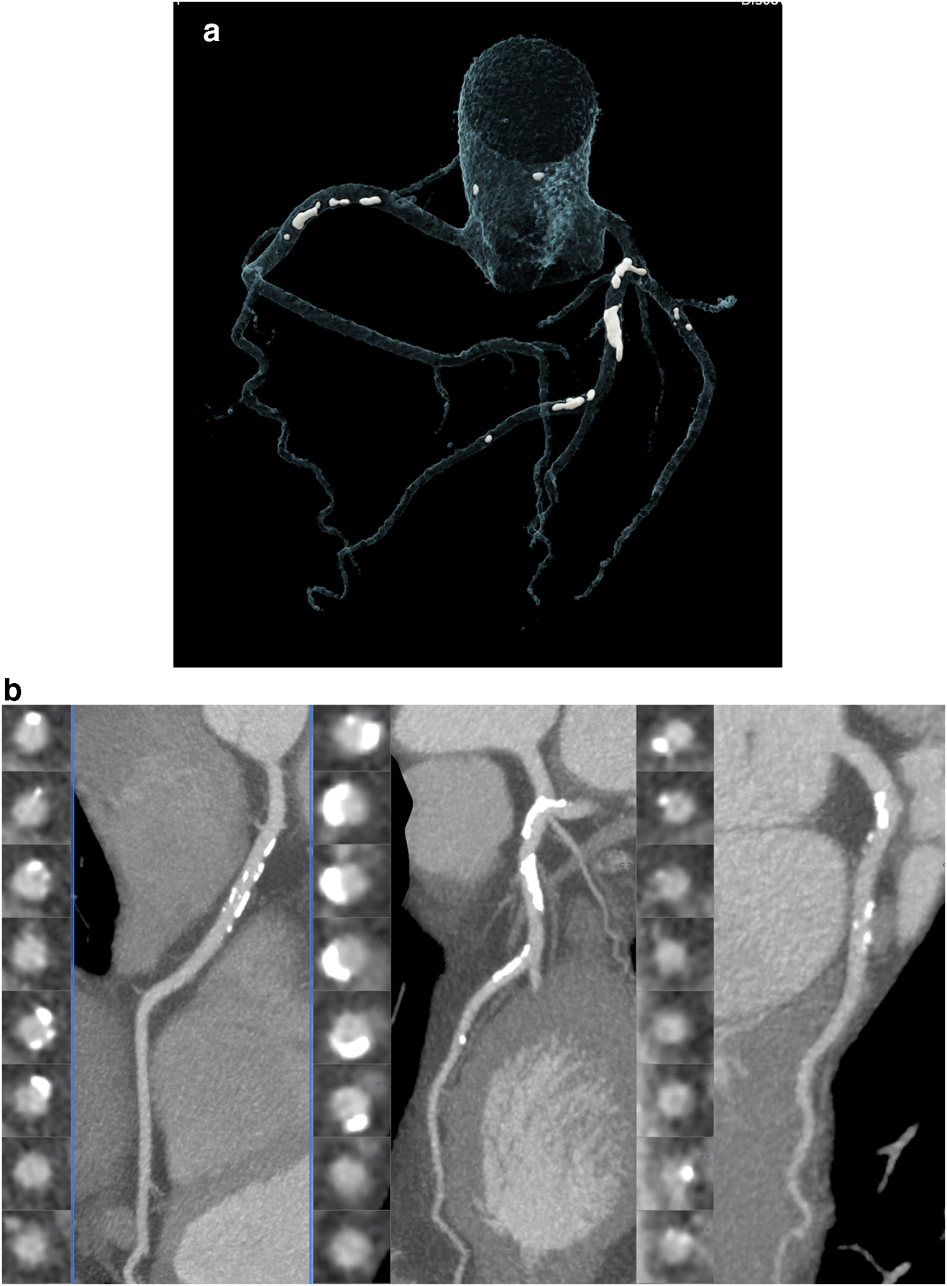
A 57-year-old-male with 1 risk factor (arterial hypertension) and ESLD due to alcohol-related liver disease. CTA was performed 2 years after quitting alcohol abuse prior to liver transplantation. MELD score was 12, and Child-Pugh Stadium B and several episodes of liver decompensations were recorded. Cardiac Calcium Score was high with 527 AU. CTA showed multiple calcified plaques in the RCA; LAD and CX with less than 50% stenosis (CAD-RADS 2). VRT (left) and cMRP (right) of RCA; LAD and CX with multiple calcified but no high-risk plaque

Figure 1 shows the coronary CTA of a former drinker with ESLD and large vessel size even at side branch and septal branch level, indicative of vasodilatory effects, and minimal plaque burden (high-density fibrous plaque). Figure 2 presents a patient with high calcium score, who quitted drinking 2 years ago.

Total mixed plaque burden was lower in patients with ESLD due to prior alcohol abuse (G-score: mean 4.9 vs. 7.4, *p* = 0.001). Figure 1 shows a patient with ESLD, low plaque burden, and a CCS of zero. Figure 2 illustrates a case of ESLD with high exclusively, calcified plaque burden.

**Fig. 3:**
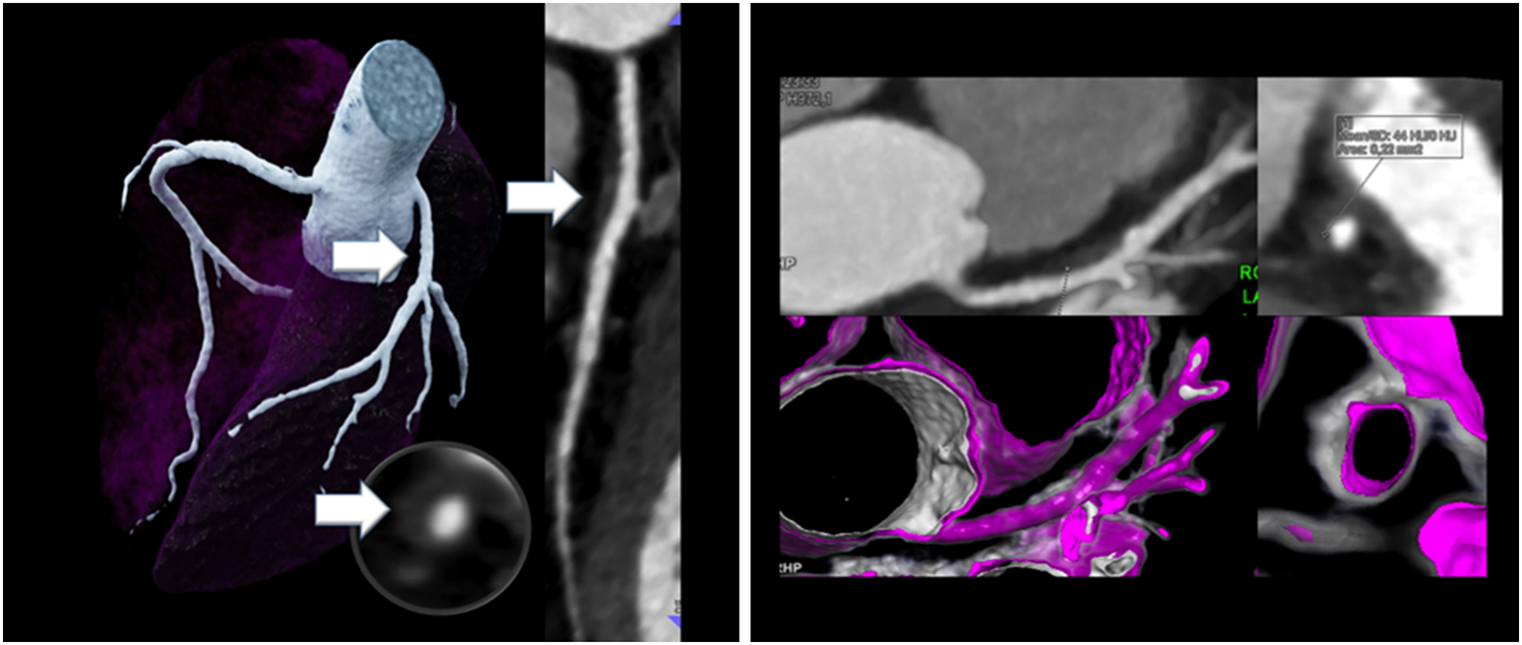
A 47-year-old-male, 1 risk factor (arterial hypertension) who denied ever drinking alcohol (control group). Low coronary calcium score (4.1 Agatston Units) but high non-calcified plaque burden (LM and LAD, white arrow) with a high-risk plaque in the proximal LAD with low attenuation (44 HU) and napkin-ring sign (inlay, outer hyperdense rim)

High-risk plaques were markedly less common in the ESLD-alc group as compared to controls (4.5% vs. 37.5%, *p* < 0.001). Also the total number of high-risk plaques was lower (Fig. 3).

Plaque density was lower in controls as compared to the ESLD-alc group (56.6 HU ± 3.2 vs. 91.3 ± 4.5), indicting more dense mixed fibro-calcific plaque burden in patients with alcoholic ESLD.

CCS was higher in ESLD patients (132.8 vs. 72.9 AU, *p* = 0.673) but the difference did not reach statistical significance. The prevalence of non-calcified plaques in patients with a CCS of zero was similar between the two groups but reached 30.7% in ESLD.

## Discussion

Our study shows lower non-calcified fibroatheroma and less high-risk plaque burden, but a trend to higher denser mixed calcified coronary plaque in patients with alcohol-related end-stage liver disease (ESLD-alc), as compared to a matched cohort without liver disease and without alcohol consumption but an identical CV risk profile.

However, total prevalence of CAD was high and similar between both groups. The reasons for our findings could be explained as follows.

None of the patients with ESLD was actively and excessively drinking alcohol at the time of coronary CTA, since they were evaluated for LT and had to be abstinent for a minimum of 6 months, in order to be listed for surgery. This may partially explain the lack of high-risk plaque as an indicator for plaque inflammation. Laboratory pro-inflammatory parameters, which are usually increased during episodes of long-term active excessive drinking, will most likely downregulate after cessation of active drinking. The distinct atherosclerosis profile (with more calcific plaque) may be also relate to other pathways driving coronary calcification: Transient or chronic kidney dysfunction is common in ESLD, which enhances coronary calcification. Repeated episodes of arterial hypertension during heavy drinking as well most likely stimulate calcification progression.

While data on CAD burden in ESLD patients are sparse, there are some studies in patients with liver disease indicating the following trends.

Both steatosis and cirrhosis of the liver have been linked to a higher CAD burden, especially to a higher calcified plaque burden [19, 20]: In 2028 patients with steatosis, a liver attenuation of < 54 HU was associated with obstructive CAD, independently of conventional cardiac risk factors. The severity of coronary calcification was associated with the severity of fatty liver [19].

In a smaller cohort of only 52 patients [20] with cirrhosis of all Child-Pugh stadiums, a high CAD prevalence (77%) was found, which is comparable to the results in our cohort. CCS and severity of CAD in terms of vessel involvement as well as the volumes of non-calcified and calcified plaques were higher [20]. In contrast to our work, this study [20] consisted of a small heterogeneous cohort of patients with cirrhosis of varying etiology.

However, until now, no study has analyzed the coronary artery disease profile a specific cohort of patients with severe ESLD due to ARLD.

As novelty, we analyzed high-risk plaque criteria [7–10], while previous studies enrolled patients with ESLD of varying etiologies, and mainly calculated only the CCS [21]. CCS is a less sensitive and less accurate method for defining the coronary atherosclerosis profile as opposed to coronary CTA: Coronary CTA allows for stratification of coronary stenosis severity (CAD-RADS), total mixed plaque burden, and plaque morphology characterization, including the detection of “high-risk” plaque criteria [18].

The effect of alcohol consumption on heart diseases is controversially discussed in the literature. While the protective effects of moderate amounts of alcohol on coronary heart disease outcomes have been well studied in epidemiological trials [4], heavy drinking has been associated with increased risk of heart failure [22] due to higher left ventricular remodeling and mass [23].

In a recent meta-analysis, even higher amounts of alcohol (> 7 drinks/day) improved outcomes in patients with heart failure [5]. However, in another meta-analysis (13 prospective studies, 355,804 participants) [24], light alcohol drinking (< 7 drinks/week) was inversely associated with risk of heart failure, while no statistically significant association between moderate (7–14 drinks/week), high (> 14 drinks/week), or heavy (> 28 drinks/week) alcohol consumption and heart failure was found. Former drinking was associated with an increased risk of heart failure compared with never or occasional drinking [24].

In a recent meta-analysis [6] including 599,912 current drinkers, alcohol consumption was roughly linearly associated with a higher risk of stroke (hazard ratio (HR) per 100 g per week higher consumption 1.14), coronary disease excluding myocardial infarction (HR 1.06), and heart failure (HR 1.09) [6].

By contrast, increased alcohol consumption was log-linearly associated with a lower risk of myocardial infarction (HR 0.94). In comparison to those who reported drinking > 0– ≤ 100 g alcohol per week, patients who drank more (> 100– ≤ 200 g per week, > 200– ≤ 350 g per week, or > 350 g per week) had lower life expectancy at age 40 of approximately 6 months (and 1–2 years, or 4–5 years, respectively).

Studies investigating the effect of alcohol on cardiovascular outcomes have shown varying results, pending on which cohorts were enrolled (patients with heart failure or healthy patients) and which endpoints were used. On a physiological level, there may be beneficial effects from antioxidants (e.g., resveratrol from red wine) and from vasodilatative effects. Hence, different types of alcohol may be more or less beneficial on the cardiovascular system. However, high levels of alcohol consumption increase blood pressure and high endothelial sheer act pro-atherogenic and stimulate the calcification process. Further, toxic interactions could also act pro-atherogenic by inducing pro-inflammatory pathways.

There is scientific evidence from epidemiologic studies (Women’s Health Study, 26,000 participants) proving that alcohol consumption is an independent predictor of cardiovascular events [4], with a J-shape (dose-dependent) association between alcohol consumption and incident cardiovascular disease (CVD) and CVD mortality. Cardioprotective effects are supposed to be modulated with anti-inflammatory mechanisms although those mechanisms have never been fully understood [4].

However, inflammatory and hemostatic factors such as C-reactive protein (CRP), soluble intracellular adhesion molecule-1, and fibrinogen were 21% lower in moderate alcohol drinkers (5–14.9 g/day) and 13% lower in heavy drinkers (> 15 g/day) compared to abstainers or occasional consumers of alcohol. Bektas et al. confirmed the absence of “inflammatory” stages of atherosclerosis [4]. This is in line with our findings, with regard to the low incidence of high-risk plaque features.

As a result, non-calcified lipid-rich plaque was found less often in drinkers as compared to “healthy” controls. Nonetheless, and surprisingly, about one third (30.7%) of patients with CCS zero had non-calcifying plaque by CTA. This finding does not support using CCS for risk stratification prior to LT.

On one hand, other comorbidities related to advanced stages of liver disease may contribute to increased coronary calcium, such as renal dysfunction. On the other hand, increased estrogen levels in patients with ESLD may have rather protective effects on atherogenesis.

We did not find a higher prevalence of signs of plaque inflammation (high-risk plaque criteria) in our populations. This may be explained by the fact that our patients quit drinking before coronary CTA as they were evaluated for LT. Thus, there were no “active” heavy drinkers at the time of coronary CTA, in contrast to the meta-analysis on “active” high alcohol consumption [6]. High-risk plaques detected by coronary CTA are predictors of adverse outcome in terms of both MACE [25] and vessel-specific ischemia [26, 27].

Interestingly, a novel study published this month showed that along with increasing plaque density (HU), the risk of MACE declines. Especially the 1-K Plaque (> above 1000 HU) had the lowest risk of MACE in the ICONIC registry and may improve risk stratification [28].

### Study limitations

We acknowledge the retrospective study design with its inherent bias. Confounding risk factors were minimized by best medical practice (1:1 propensity score matching).

All patients had a history of previous excessive alcohol consumption, resulting in ESLD. Because they were evaluated for LT, they had to prove to be abstinent by regular controls of their urine ethyl glucuronide levels for at least 6 months and regular psychological evaluation.

The time between alcohol renouncement and CTA varied, since the time interval between quitting and coronary CTA ranged from a minimum of 6 months up to several years (> 6 years).

Of note, we would like to emphasize, that the CAD disease profile in our cohort is specific for ESLD patients prior to LT due to alcohol abuse, but may be distinct in patients with alcohol abuse without ESLD.

We acknowledge that a sub-analysis of the type of alcohol consumption cannot be provided due to the mixed drinking behavior and incomplete data collection.

Hard study endpoints (MACE; mortality) were not collected. Therefore, a conclusion, whether calcium scoring or coronary CTA is better for risk stratification prior to LT, is not possible. However, the high total prevalence of CAD in the ESDL cohort rather suggests the use of CTA, in order to identify individuals with significant stenosis, who require invasive coronary angiography and coronary intervention in order to avoid adverse outcomes after LT and intraoperative cardiovascular complications during LT. Coronary CTA has shown to be superior to CCS for CV risk stratification in numerous data outputs from CONFIRM registry [29, 30].

## Conclusion

Patients with alcohol-related ESLD exhibit have a high total prevalence of CAD with a higher calcified plaque burden but less high-risk “vulnerable” plaque characteristics and a less lipid-rich plaque burden. Patients with ESLD have poor cardiovascular outcomes [31].

Our study sheds light on the mechanism of alcohol-related ESLD on the coronary arteries, in terms of reducing “high-risk” inflammatory plaque but increasing coronary calcification, while maintaining an overall high CAD burden.

In summary, our data support using CTA rather than calcium scoring for the evaluation of patients with ESLD prior to LT. The advantages of CTA comprise: Detection of CAD at early stages and adverse plaque characteristic [32], and diagnosis of significant stenosis [29, 30], and to initiate appropriate treatment for prevention of adverse cardiovascular outcomes, during and after liver transplantation, a high-risk surgical procedure.

## Abbreviations

ACS: Acute coronary syndrome
AHA: American Heart Association
ARLD: Alcohol-related liver disease
AU: Agatston units
BMI: Body mass index
CABG: Coronary artery bypass graft
CAD: Coronary artery disease
CAD-RADS: Coronary Artery Disease–Reporting and Data System
CCS: Coronary calcium score
cMPR: Curved multiplanar reformations
CRP: C-reactive protein
CTA: Computed tomography angiography
CVD: Cardiovascular disease
EF: Ejection fraction
ESLD: End-stage liver disease
GFR: Glomerular filtration rate
HR: Hazard ratio
HU: Hounsfield units
INR: International normalized ratio
LAP: Low attenuating plaque
LT: Liver transplantation
LVEF: Left ventricular ejection fraction
MACE: Major adverse cardiac event
MELD: Model for end-stage liver disease
NRS: Napkin-ring sign
NCP: Non-calcified plaque
RI: Remodeling index
ROI: Region of interest
SD: Standard deviation

## References

[1] VanWagner LB, Lapin B, Levitsky J (2014). High early cardiovascular mortality after liver transplantation. Liver Transpl.

[2] Kong YG, Kang JW, Kim YK (2015). Preoperative coronary calcium score is predictive of early postoperative cardiovascular complications in liver transplant recipients. Br J Anaesth.

[3] Jodocy D, Abbrederis S, Graziadei IW (2012). Coronary computer tomographic angiography for preoperative risk stratification in patients undergoing liver transplantation. Eur J Radiol.

[4] Bektas A, Sen R, Ferrucci L (2016). Does a bit of alcohol turn off inflammation and improve health?. Age Ageing.

[5] Sadhu JS, Novak E, Mukamal KJ (2018). Association of alcohol consumption after development of heart failure with survival among older adults in the cardiovascular health study. JAMA Netw Open.

[6] Wood AM, Kaptoge S, Butterworth AS (2018). Risk thresholds for alcohol consumption: combined analysis of individual-participant data for 599 912 current drinkers in 83 prospective studies. Lancet.

[7] SCOT-HEART Investigators, Newby DE, Adamson PD et al (2018) Coronary CT angiography and 5-year risk of myocardial infarction. N Engl J Med 379:924–933 10.1056/NEJMoa1805971 30145934

[8] Thomsen C, Abdulla J (2016). Characteristics of high-risk coronary plaques identified by computed tomographic angiography and associated prognosis: a systematic review and meta-analysis. Eur Heart J Cardiovasc Imaging.

[9] Maurovich-Horvat P, Schlett CL, Alkadhi H (2012). The napkin-ring sign indicates advanced atherosclerotic lesions in coronary CT angiography. JACC Cardiovasc Imaging.

[10] Feuchtner G, Kerber J, Burghard P (2017). The high-risk criteria low-attenuation plaque <60 HU and the napkin-ring sign are the most powerful predictors of MACE: a long-term follow-up study. Eur Heart J Cardiovasc Imaging.

[11] Kim HJ, Lee HW (2013). Important predictor of mortality in patients with end-stage liver disease. Clin Mol Hepatol.

[12] American Psychiatric Association (2013) Diagnostic and statistical manual of mental disorders. 10.1176/appi.books.9780890425596.dsm16

[13] European Association for the Study of the Liver (2018) EASL clinical practice guidelines: management of alcohol-related liver disease. J Hepatol 69:154–181 10.1016/j.jhep.2018.03.018 29628280

[14] Agatston AS, Janowitz WR, Hildner FJ, Zusmer NR, Viamonte M, Detrano R (1990). Quantification of coronary artery calcium using ultrafast computed tomography. J Am Coll Cardiol.

[15] Cury RC, Abbara S, Achenbach S (2016). CAD-RADS(TM) Coronary Artery Disease - Reporting and Data System. An expert consensus document of the Society of Cardiovascular Computed Tomography (SCCT), the American College of Radiology (ACR) and the North American Society for Cardiovascular Imaging (NASCI). Endorsed by the American College of Cardiology. J Cardiovasc Comput Tomogr.

[16] Austen WG, Edwards JE, Frye RL (1975). A reporting system on patients evaluated for coronary artery disease. Report of the Ad Hoc Committee for Grading of Coronary Artery Disease, Council on Cardiovascular Surgery, American Heart Association. Circulation.

[17] Feuchtner GM, Barbieri F, Langer C et al (2019) Non obstructive high-risk plaque but not calcified by coronary CTA, and the G-score predict ischemia. J Cardiovasc Comput Tomogr. 10.1016/j.jcct.2019.01.010 10.1016/j.jcct.2019.01.010 30661963

[18] Leber AW, Knez A, Becker A (2004). Accuracy of multidetector spiral computed tomography in identifying and differentiating the composition of coronary atherosclerotic plaques: a comparative study with intracoronary ultrasound. J Am Coll Cardiol.

[19] Tomizawa N, Inoh S, Nojo T, Nakamura S (2016). Relationship of hepatic steatosis severity and coronary artery disease characteristics assessed by coronary CT angiography. Int J Cardiovasc Imaging.

[20] Kazankov K, Munk K, Ovrehus KA (2017). High burden of coronary atherosclerosis in patients with cirrhosis. Eur J Clin Invest.

[21] McAvoy NC, Kochar N, McKillop G, Newby DE, Hayes PC (2008). Prevalence of coronary artery calcification in patients undergoing assessment for orthotopic liver transplantation. Liver Transpl.

[22] Mouton AJ, Ninh VK, El Hajj EC, El Hajj MC, Gilpin NW, Gardner JD (2016). Exposure to chronic alcohol accelerates development of wall stress and eccentric remodeling in rats with volume overload. J Mol Cell Cardiol.

[23] Rodrigues P, Santos-Ribeiro S, Teodoro T (2018). Association between alcohol intake and cardiac remodeling. J Am Coll Cardiol.

[24] Larsson SC, Wallin A, Wolk A (2018). Alcohol consumption and risk of heart failure: meta-analysis of 13 prospective studies. Clin Nutr.

[25] Hell MM, Motwani M, Otaki Y (2017). Quantitative global plaque characteristics from coronary computed tomography angiography for the prediction of future cardiac mortality during long-term follow-up. Eur Heart J Cardiovasc Imaging.

[26] Rizvi A, Hartaigh BO, Danad I (2017). Diffuse coronary artery disease among other atherosclerotic plaque characteristics by coronary computed tomography angiography for predicting coronary vessel-specific ischemia by fractional flow reserve. Atherosclerosis.

[27] Ahmadi A, Leipsic J, Ovrehus KA (2018). Lesion-specific and vessel-related determinants of fractional flow reserve beyond coronary artery stenosis. JACC Cardiovasc Imaging.

[28] van Rosendael AR, Narula J, Lin FY et al (2020) Association of high-density calcified 1K Plaque with risk of acute coronary syndrome. JAMA Cardiol. 10.1001/jamacardio.2019.5315 10.1001/jamacardio.2019.5315 PMC6990946 31968065

[29] Xie JX, Cury RC, Leipsic J (2018). The Coronary Artery Disease-Reporting and Data System (CAD-RADS): prognostic and clinical implications associated with standardized coronary computed tomography angiography reporting. JACC Cardiovasc Imaging.

[30] Han D, Hartaigh BO, Gransar H (2018). Incremental prognostic value of coronary computed tomography angiography over coronary calcium scoring for major adverse cardiac events in elderly asymptomatic individuals. Eur Heart J Cardiovasc Imaging.

[31] Ripoll C, Yotti R, Bermejo J, Banares R (2011). The heart in liver transplantation. J Hepatol.

[32] Williams MC, Moss AJ, Dweck M (2019). Coronary artery plaque characteristics associated with adverse outcomes in the SCOT-HEART study. J Am Coll Cardiol.

